# Exploring water, sanitation, and hygiene coverage targets for reaching and sustaining trachoma elimination: G-computation analysis

**DOI:** 10.1371/journal.pntd.0011103

**Published:** 2023-02-13

**Authors:** Kristin M. Sullivan, Emma M. Harding-Esch, Alexander P. Keil, Matthew C. Freeman, Wilfrid E. Batcho, Amadou A. Bio Issifou, Victor Bucumi, Assumpta L. Bella, Emilienne Epee, Segni Bobo Barkesa, Fikre Seife Gebretsadik, Salimato Sanha, Khumbo M. Kalua, Michael P. Masika, Abdallahi O. Minnih, Mariamo Abdala, Marília E. Massangaie, Abdou Amza, Boubacar Kadri, Beido Nassirou, Caleb D. Mpyet, Nicholas Olobio, Mouctar D. Badiane, Balgesa E. Elshafie, Gilbert Baayenda, George E. Kabona, Oscar Kaitaba, Alistidia Simon, Tawfik Q. Al-Khateeb, Consity Mwale, Ana Bakhtiari, Daniel Westreich, Anthony W. Solomon, Emily W. Gower

**Affiliations:** 1 Department of Epidemiology, University of North Carolina at Chapel Hill, Chapel Hill, North Carolina, United States of America; 2 Clinical Research Department, London School of Hygiene & Tropical Medicine, London, United Kingdom; 3 Gangarosa Department of Environmental Health, Emory University, Atlanta, Georgia, United States of America; 4 Programme National De Lutte Contre Les Maladies Transmissibles, Ministère De La Santé, Cotonou, Benin; 5 Département D’ophthalmologie, Université De Parakou, Parakou, Borgou, Benin; 6 Département En Charge des Maladies Tropicales, Négligées Ministère De La Santé Publique Et De La Lutte Contre Le Sida, Bujumbura, Burundi; 7 Programme National De Lutte Contre La Cécité, Ministère De La Santé Publique, Yaounde, Cameroon; 8 Department Of Ophthalmology, University of Yaoundé 1 Yaounde Centre, Yaoundé, Cameroun; 9 Neglected Tropical Disease Prevention and Control Program, Federal Ministry of Health, Addis Ababa, Ethiopia; 10 Programa Nacional De Saúde De Visão, Minsap, Bissau, Guinea-Bissau; 11 Blantyre Institute for Community Outreach, Blantyre, Malawi; 12 Department of Clinical Services, Ministry of Health, Lilongwe, Malawi; 13 Département Des Maladies Transmissibles, Ministère De La Santé Nouakchott, Nouakchott, Mauritania; 14 Direcção Nacional De Saúde Pública Ministerio Da Saude, Maputo, Mozambique; 15 Programme National De Santé Oculaire Ministère De La Santé Publique, Niamey, Niger; 16 Department of Ophthalmology, University of Jos, Jos, Plateau, Nigeria; 17 Neglected Tropical Diseases Division, Department of Public Health, Federal Ministry of Health, Abuja, Nigeria; 18 Programme National de Promotion de La Santé Oculaire, Ministère de la Santé et de L’Action sociale, Dakar, Senegal; 19 National Program for Prevention of Blindness, Federal Ministry of Health, Khartoum, Sudan; 20 National Disease Control, Ministry of Health, Kampala, Uganda; 21 Neglected Tropical Disease Control Program, Ministry of Health, Dodoma, United Republic of Tanzania; 22 Department Of Ophthalmology, Sana’a University, Sana’a, Yemen; 23 Provincial Health Office, Ministry of Health, Lusaka, Zambia; 24 International Trachoma Initiative, Task Force for Global Health, Decatur, Georgia, United States of America; 25 Department of Control of Neglected Tropical Diseases, World Health Organization, Geneva, Switzerland; Universidade do Estado do Rio de Janeiro, BRAZIL

## Abstract

**Background:**

Trachoma is the leading infectious cause of blindness. To reduce transmission, water, sanitation, and hygiene (WaSH) improvements are promoted through a comprehensive public health strategy. Evidence supporting the role of WaSH in trachoma elimination is mixed and it remains unknown what WaSH coverages are needed to effectively reduce transmission.

**Methods/Findings:**

We used g-computation to estimate the impact on the prevalence of trachomatous inflammation—follicular among children aged 1–9 years (TF_1-9_) when hypothetical WaSH interventions raised the minimum coverages from 5% to 100% for “nearby” face-washing water (<30 minutes roundtrip collection time) and adult latrine use in an evaluation unit (EU). For each scenario, we estimated the generalized prevalence difference as the TF_1-9_ prevalence under the intervention scenarios minus the observed prevalence. Data from 574 cross-sectional surveys conducted in 16 African and Eastern Mediterranean countries were included. Surveys were conducted from 2015–2019 with support from the Global Trachoma Mapping Project and Tropical Data.

When modeling interventions among EUs that had not yet met the TF_1-9_ elimination target, increasing nearby face-washing water and latrine use coverages above 30% was generally associated with consistent decreases in TF_1-9_. For nearby face-washing water, we estimated a ≥25% decrease in TF_1-9_ at 65% coverage, with a plateau upon reaching 85% coverage. For latrine use, the estimated decrease in TF_1-9_ accelerated from 80% coverage upward, with a ≥25% decrease in TF_1-9_ by 85% coverage. Among EUs that had previously met the elimination target, results were inconclusive.

**Conclusions:**

Our results support Sustainable Development Goal 6 and provide insight into potential WaSH-related coverage targets for trachoma elimination. Targets can be tested in future trials to improve evidence-based WaSH guidance for trachoma.

## Author summary

Previous work has been unable to determine what water, sanitation, and hygiene (WaSH)-related coverages are needed to optimally limit trachoma transmission. This study uses a large, multi-national dataset to explore the impact of hypothetical WaSH interventions designed to increase coverages of face-washing water and latrine use in districts that have met and those that have not met trachoma elimination targets. We used statistical models to explore how these interventions impacted the prevalence of trachoma among children as compared to the observed data.

Our findings provide evidence-based insight into potential WaSH coverage targets that could be hypothesized to achieve meaningful reductions in trachoma prevalence. We found that in areas working to reach trachoma elimination targets, increasing face-washing water and latrine use coverages to a minimum of ≥30% were consistently associated with (modelled) reductions in active trachoma prevalence. However, in areas that had already met trachoma elimination targets, we did not see the same pattern. This finding supports our theory that the WaSH-trachoma relationship differs in these areas and suggests a need for additional research to explore these relationships. Our estimates can be used to inform programmatic WaSH targets and future field trials.

## Introduction

Trachoma is the leading infectious cause of blindness. Visual impairment due to trachoma results from repeated infections with *Chlamydia trachomatis*. Approximately 136 million people live in trachoma-endemic districts of 44 countries, and 1.8 million people need surgery to prevent trachomatous blindness [1,2].

To reduce *C*. *trachomatis* transmission, environmental improvement is recommended as part of the "SAFE" strategy (Surgery, Antibiotics, Facial cleanliness, and Environmental improvement). The "E" component involves improved access to water, sanitation, and hygiene (WaSH) to reduce eye and nose secretions and human feces in the environment (the latter being the preferred breeding site for the vector, the *Musca sorbens* fly). The "E" component is delivered at the evaluation unit (EU) level, defined by the World Health Organization (WHO) as the "administrative unit for healthcare management consisting of a population unit between 100,000–250,000 persons [3]." The success of SAFE interventions in making progress toward eliminating trachoma as a public health problem is monitored using the EU-level prevalence of trachomatous inflammation—follicular (TF) among 1–9-year-olds (TF_1-9_) [4,5].

Despite the success of SAFE interventions, over 1,300 EUs have not yet met trachoma elimination targets [2]. This suggests a need to optimize SAFE and its delivery. Presently, guidance on "E" suggests only that programs work with community partners to increase WaSH availability [6]. Current evidence has not identified minimum WaSH coverages for effectively reducing transmission. Furthermore, though EUs are reaching elimination targets and annual mass drug administration (MDA) is being discontinued, no WaSH studies have been conducted specifically in the post-MDA context. The nature of the WaSH-trachoma relationship remains unclear and optimal strategies for the prevention of trachoma via WaSH have not been determined.

To inform programmatic targets and future field trials, we used data collected during uniformly-conducted surveys from 16 countries to explore population-level impacts of hypothetical WaSH interventions on TF_1-9_. Our objective was to estimate differences between TF_1-9_ from existing WaSH coverage distributions and TF_1-9_ when hypothetical interventions raised coverages of nearby face-washing water and latrine use. We separately analyzed EUs that had not yet met the elimination target (TF_1-9_ <5%) and EUs that had met this target.

## Methods

### Ethics statement

The ethics committee at the University of North Carolina at Chapel Hill determined that this study did not constitute human subjects research (18–2360). It was approved by the ethics committee at the London School of Hygiene & Tropical Medicine (16275). GTMP/TD examiners sought informed consent before surveying. Data were collected, transmitted, and stored in a manner intended to protect participant anonymity and confidentiality.

### Primary data source

Primary data were collected in population-based surveys supported by the Global Trachoma Mapping Project (GTMP; December 2012–January 2016; 29 countries) and Tropical Data (TD; initiated February 2016; 46 countries to date). These surveys are conducted where trachoma is or was suspected to be endemic. Comprehensive methodological details have been published elsewhere [3,7], however key details are provided below.

#### GTMP/TD survey design

Surveys are conducted at the EU level using two-stage cluster sampling, with sampling at the village and household levels [7]. Sampling is designed such that, to the extent possible, all EU residents have equal selection probability. All household members aged ≥1 year are eligible to participate.

#### GTMP/TD survey implementation

In each selected household, consenting residents are examined by certified trachoma graders after consent is obtained [8]. Graders evaluate each eye for TF based on the WHO trachoma simplified grading scheme [5,9]. TF is indicated by the presence of ≥5 follicles, each ≥0.5 mm in diameter, in the central part of the upper tarsal conjunctiva. People with active trachoma are offered antibiotic treatment.

WaSH survey questions are asked at the household level and are typically answered by self-nominated heads of households. WaSH questions, developed in line with WHO/UNICEF Joint Monitoring Program indicators, ask (1) the source and distance to water used for drinking during the dry season, (2) the source type and distance to water used for washing faces during the dry season, (3) the latrine types used and defecation practices among adults, and (4) the handwashing facilities available.

All data, including household GPS location, are captured electronically, and uploaded to a server.

#### Survey purpose and nomenclature

Three survey types are conducted for estimating TF_1-9_. *Baseline surveys* are conducted in suspected-endemic EUs to establish baseline prevalence. After completing the recommended 1–5 years of AFE interventions, *impact surveys* are conducted to determine if the EU has reached a TF_1-9_ <5%. If that target is not met, interventions continue. If the target is met, the EU discontinues MDA (F and E continue). A surveillance survey is conducted two years later to determine if TF_1-9_ remains <5%.

### Present analysis

#### Setting

We invited all countries that had surveyed with GTMP/TD support through 2019 to share data. EUs that had been surveyed at least twice in the baseline-impact, impact-impact, or impact-surveillance survey sequence were eligible. Most EU boundaries did not change over time. If they did, we included an EU if its entire area was contained within a single prior EU boundary.

#### Design

Using data from the most recent survey in the EU, we estimated generalized intervention prevalence differences for two hypothetical WaSH exposures achieving serially increasing coverage levels. The generalized intervention prevalence difference contrasts the observed prevalence with the prevalence observed in a population in which, counter to the fact, there was increased exposure coverage due to a hypothetical yet realistic intervention [10]. In this study, the term "intervention" describes any hypothetical approaches (e.g., technological, behavioral, etc.) that would achieve the desired WaSH coverage in an EU. To correspond with programmatic activities, the EU was our chosen unit of analysis, rather than smaller geographic units such as clusters.

Our hypothetical interventions were operationalized by identifying EUs with coverage below a target and then predicting overall prevalence, had those EUs been brought up to the coverage target. This approach implies that, as the intervention level increases, increasingly more EUs are intervened upon, so it is naturally dependent on the distribution of exposures in the population and respects the idea that the positive impacts of a realistic intervention would have natural bounds determined by how much the population is exposed prior to the intervention (unlike standard regression estimates which typically contrast “everyone exposed” vs. “no-one exposed”).

#### EU categorization

We categorized eligible EUs by their presumed programmatic goal over the survey period: "reaching elimination target" EUs had a prior baseline or impact survey and were seeking to reach TF_1-9_ <5% at the subsequent impact survey; "maintaining elimination target" EUs had TF_1-9_ <5% at a prior impact survey and were seeking to demonstrate that TF_1-9_ had remained <5% at the subsequent surveillance survey. This distinction stratified the sample into EUs in which MDA *was* and *was not* recommended over the observation period. If ≥3 surveys were conducted in an EU and the most recent survey was a surveillance survey (e.g., baseline-impact-surveillance), the surveillance survey data were used in the maintaining elimination target EUs analysis, and the impact survey data were used in the reaching elimination target EUs analysis.

#### Population

**Fig 1:**
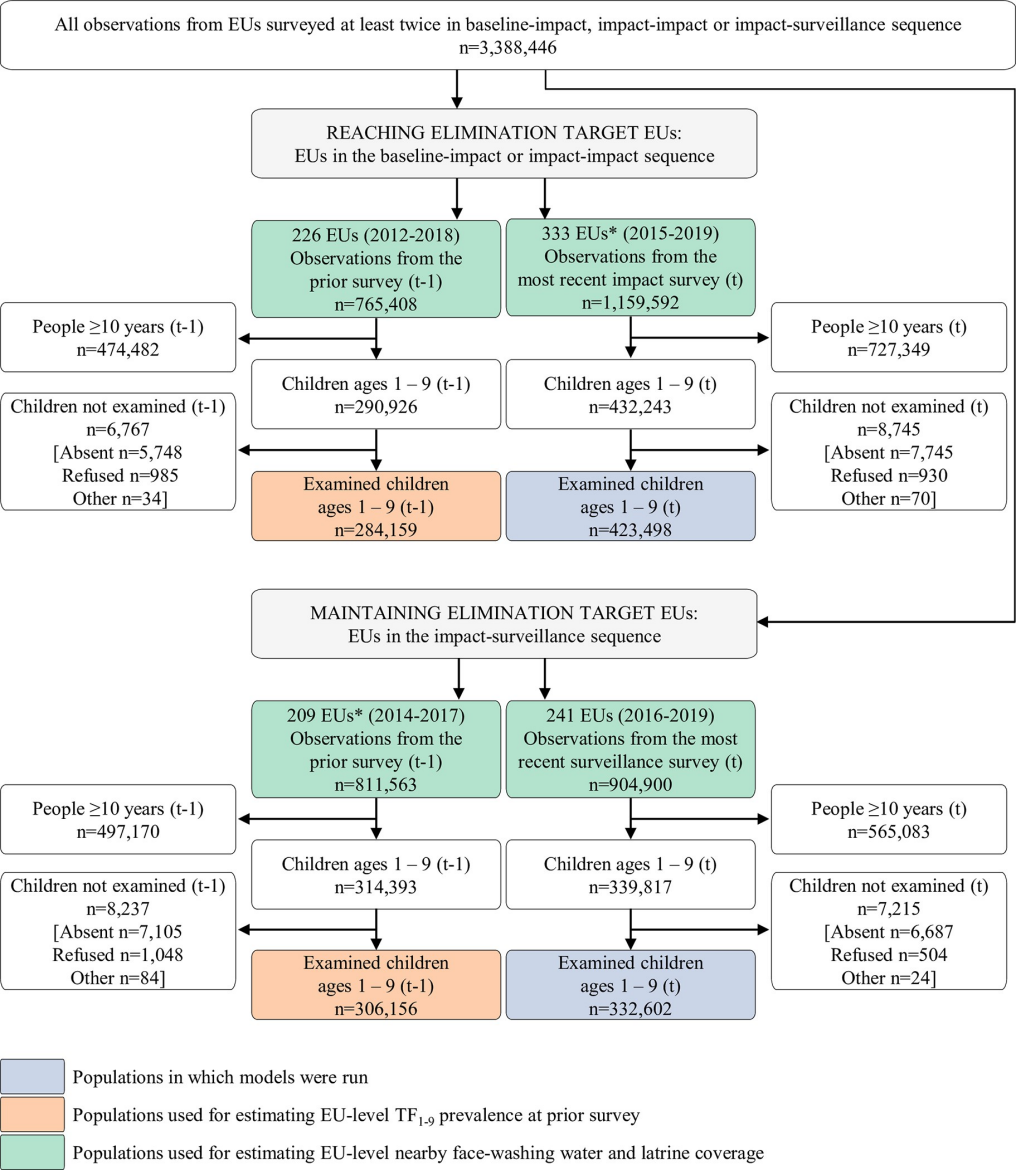
EU and participant inclusion by elimination group. Observations from the most recent surveys (denoted with a t) were the basis for the study populations, while observations from the prior surveys (denoted with a t-1) were used to calculate EU-level measures used for confounding adjustments. 69 EUs (n = 253,017 individuals) had a survey sequence of baseline-impact-surveillance and were therefore included in both the reaching elimination target EUs and in the adjustment EUs for the maintaining elimination target EUs.

The study population comprised 1–9-year-olds examined during the most recent survey in each EU (Fig 1, blue boxes).

#### Outcome, exposures, covariates

The model outcome was participant-level presence or absence of TF in either eye.

We characterized WaSH-related exposures in two household-level and two EU-level ways. The first household-level exposure was the collection time for face-washing water used during the dry season (the period during which washing water may be least available). For each household, we defined "nearby" face-washing water as water requiring <30 minutes roundtrip collection time and "not nearby" as ≥30 minutes roundtrip collection time. This facilitated comparison with prior literature and aligned with WHO/UNICEF Joint Monitoring Programme drinking water program indicators (where washing water indicators are not used) [11]. We did not differentiate between improved and unimproved water sources, as this distinction is less crucial for trachoma where water quantity is believed to be more important.

The second household-level exposure categorized defecation sites of adult householders. We assigned "latrine use" to households in which adults usually defecated in latrines (private, shared, or public) or other closed/potentially closed sites (e.g., chamber pots, buckets, or surface water) and "no latrine use" to other households (defecation outside in open, exposed environments). We used the label "latrine use" because only 0.3% of households reported non-latrine closed facility use. We did not distinguish between improved and unimproved latrines because *M*. *sorbens* preferentially oviposits on human feces left exposed on the soil in open environments [12–14].

We characterized exposures at the EU level as (1) the proportion of households that reported nearby face-washing water use ("nearby face-washing water coverage") and (2) the proportion of households that reported usual latrine use by adults ("latrine coverage"). Coverages were estimated from all surveyed households in the EU, including those with and without resident children (Fig 1, green boxes).

We constructed a directed acyclic graph (S1 Fig) based on relationships established through previous studies to determine potential confounders [15]. We included covariates at EU, household, and individual levels. Environmental covariates were considered but were not part of the minimally sufficient adjustment set. Since surveyed households and individuals differed for each of the paired EU surveys, data from the prior survey in the EU was only used to adjust for EU-level confounding. EU-level confounders were: TF_1-9_ at the prior survey (Fig 1, orange boxes), nearby face-washing water coverage at the prior survey, latrine coverage at the prior survey, time between end of the prior survey and start of the most recent survey, and country. The only household-level confounder was population density (people/km^2^) surrounding the household. This was extracted from external georeferenced raster data for the survey year using unconstrained estimates from 2015–2019 (worldpop.org). Child’s age was included as a covariate.

#### Statistical methods

We used g-computation [16,17] to estimate the impacts of hypothetical increases in EU-level nearby face-washing water and latrine coverages. We chose G-computation because it allowed us to extend traditional modeling approaches (which typically compare the complete absence to the complete presence of an exposure) to a framework that allows for comparisons of specific interventions of interest (e.g., shifting latrine coverage up from an existing distribution). Generalized intervention effects estimated from this approach were most closely tied to our objective of identifying EU-level coverages needed to prevent trachoma.

In this study, G-computation involved: 1) fitting a standard statistical model for individual-level TF that included exposures and covariates, and 2) using the statistical model parameters to estimate the TF_1-9_ distribution expected under hypothetical WaSH interventions (see S2 Fig for additional detail).

For each elimination group, we developed a logistic hierarchical model with random intercepts at EU and cluster levels (household-level random effects being too computationally resource-intensive to model). We ran models among 1–9-year-olds examined during the most recent survey. To account for potential non-linearity, all continuous exposures and covariates were modeled using restricted quadratic splines with knots at the 5^th^, 50^th^, and 95^th^ percentiles of the case distribution [18]. We selected the model with overall best fit, accounting for both groups, based on the Akaike information criterion values compared across forms, spline types, and knot locations.

We implemented hypothetical WaSH interventions, achieving intervention coverages that began at 5% and increased in 5-unit increments. We contrasted three scenarios: intervention on nearby face-washing water coverage only, intervention on latrine coverage only, and both interventions simultaneously (at the same minimum coverage levels).

To account for the fact that supplying water to a single household through a community well, for example, would also make water available for nearby households, the hypothetical face-washing water intervention was delivered at cluster level. No intervention was delivered if the EU face-washing water coverage of the observed data at the most recent survey was at or above the minimum coverage target. If it was below the minimum coverage target, a cluster was randomly selected and all households within that cluster were hypothetically provided face-washing water. Clusters were randomly selected to receive the intervention until the minimum coverage target was reached. The hypothetical latrine use intervention was delivered at household level. If the EU latrine coverage of the observed data was at or above the minimum coverage target, no intervention was delivered. If it was below the minimum coverage target, unexposed households were randomly selected to receive the hypothetical intervention with probability equal to the difference between the observed coverage and the target coverage.

Next, we used parameters generated from the statistical model to estimate the predicted probability of TF for each child under the series of hypothetical interventions implemented at the EU level. We compared the observed data to each hypothetical intervention scenario using predicted probabilities. At each minimum coverage target, we estimated the generalized intervention prevalence difference as the mean predicted probability from the hypothetical intervention scenario minus the mean predicted probability from the observed data (see https://github.com/alexpkeil1/WaSH_Int for example code).

We estimated 95% confidence intervals using cluster bootstrapping (with sampling following the original survey design); limits were the 2.5^th^ and 97.5^th^ percentiles at each minimum coverage target from a distribution of 1,000 replicates.

Analyses were conducted using SAS version 9.04.01M5 (Cary, NC). We used ArcMap version 10.8.1 (Redlands, CA) to extract point values from raster data.

## Results

### Study EUs and participants

Of 42 countries invited to participate, 32 provided data. Sixteen countries were included, all in WHO’s African and Eastern Mediterranean regions. These countries contributed 574 eligible surveys with a paired prior survey. Fifteen countries were ineligible because there were no paired surveys in any EU. One eligible survey from a South Pacific country was excluded because it was the only eligible survey with a paired prior survey in a region where trachoma epidemiology is unusual [19].

Among 574 eligible surveys, 333 EUs (261,899 households) were included in the reaching elimination target EUs and 241 EUs (200,401 households) in the maintaining elimination target EUs (Table 1). TF_1-9_ was <5% in both groups: 4.7% (95% survey-design corrected CI: 4.7%-4.8%) among 423,498 examined children in the reaching elimination target EUs and 2.1% (95% survey-design corrected CI: 2.0%-2.1%) among 332,602 examined children in the maintaining elimination target EUs. Overall, ~52% of households reported using nearby face-washing water in both groups. Household latrine use by adults was ~16 percentage points higher in the maintaining elimination target EUs (81.0%) than in the reaching elimination target EUs (64.9%).

**Table 1:** Characteristics of study EUs, clusters, households, and individuals.

	EUs	Clusters	Households					Indivi-duals	Children aged 1–9 years old			
	Surveyed	Surveyed	Surveyed	Using nearby face-washing water*	Missing face-washing water exposure	Using latrines†	Missing latrine use exposure	Surveyed	Surveyed	Examined	Examined children with TF^‡^	Examined, but missing TF status
**Reaching elimination target EUs** ^§^												
Benin	8	192	4,923	2,790 (56.7%)	0	805 (16.4%)	0	24,636	12,519	12,480	127 (1.0%)	0
Burundi	1	23	697	273 (39.5%)	5	679 (97.4%)	0	3,351	1,327	1,303	40 (3.1%)	0
Ethiopia	155	4,150	126,030	56,960 (45.2%)	31	70,510 (55.9%)	0	522,421	171,391	168,920	12,463 (7.4%)	13
Malawi	34	852	26,242	13,474 (51.3%)	0	24,163 (92.1%)	0	117,858	38,867	36,365	663 (1.8%)	2
Mozambique	23	606	21,108	7,480 (35.4%)	0	9,354 (44.3%)	0	83,791	35,252	34,151	1,197 (3.5%)	0
Nigeria	51	1,285	32,142	24,955 (77.7%)	31	24,956 (77.6%)	0	176,164	78,781	77,262	1,886 (2.4%)	4
Senegal	11	327	9,791	9,598 (98.0%)	0	9,351 (95.5%)	0	46,398	17,666	17,550	330 (1.9%)	0
Sudan	4	100	2,994	1,729 (57.7%)	0	1,704 (56.9%)	0	12,080	5,758	5,659	197 (3.5%)	0
Tanzania	21	550	19,457	6,820 (35.1%)	8	15,639 (80.4%)	0	89,821	37,261	36,758	1,768 (4.8%)	1
Uganda	9	232	7,212	2,095 (29.0%)	0	3,687 (51.1%)	0	32,148	15,293	15,118	553 (3.7%)	6
Yemen	4	80	2,417	1,876 (77.6%)	0	1,948 (80.6%)	0	11,682	3,825	3,811	171 (4.5%)	1
Zambia	12	288	8,886	7,680 (86.4%)	0	7,056 (79.4%)	0	39,242	14,303	14,121	665 (4.7%)	1
**Total**	**333**	**8,685**	**261,899**	**135,730 (51.8%)**	**75**	**169,852 (64.9%)**	**0**	**1,159,592**	**432,243**	**423,498**	**20,060 (4.7%)**	**28**
**Maintaining elimination target EUs** ^‖^												
Cameroon	11	288	8,751	5,667 (64.8%)	0	6,506 (74.3%)	0	36,194	12,975	12,922	455 (3.5%)	0
Ethiopia	19	544	16,360	8,456 (51.7%)	0	7,836 (47.9%)	0	65,614	18,211	17,707	945 (5.3%)	9
Guinea Bissau	5	108	3,237	2,969 (91.7%)	0	2,901 (89.6%)	0	16,735	5,644	5,537	157 (2.8%)	0
Malawi	38	1,044	31,634	17,113 (54.1%)	0	28,670 (90.6%)	0	138,661	44,719	43,395	707 (1.6%)	1
Mauritania	6	143	4,283	3,677 (85.9%)	0	3,333 (77.8%)	0	18,085	6,871	6,867	34 (0.5%)	0
Mozambique	23	552	19,250	5,169 (27.2%)	263	11,923 (61.9%)	0	78,028	33,244	32,049	975 (3.0%)	0
Niger	3	90	2,699	1,897 (70.3%)	0	666 (24.7%)	0	15,327	7,496	7,477	245 (3.3%)	0
Nigeria	14	355	8,890	7,076 (79.6%)	0	5,974 (67.2%)	0	46,277	20,536	19,971	517 (2.6%)	0
Senegal	21	630	18,901	18,519 (98.0%)	0	16,902 (89.4%)	0	92,734	36,116	35,829	515 (1.4%)	0
Tanzania	53	1,400	48,900	19,881 (40.7%)	13	44,224 (90.4%)	0	218,747	81,261	79,089	1,462 (1.8%)	8
Uganda	42	1,084	33,111	10,377 (31.3%)	0	29,789 (90.0%)	0	157,650	65,159	64,283	685 (1.1%)	36
Zambia	6	143	4,385	3,366 (76.8%)	0	3,540 (80.7%)	0	20,848	7,585	7,476	265 (3.5%)	0
**Total**	**241**	**6,381**	**200,401**	**104,167 (52.1%)**	**276**	**162,264 (81.0%)**	**0**	**904,900**	**339,817**	**332,602**	**6,962** **(2.1%)**	**54**

Figures shown are n or n (% of non-missing responses). EU: Evaluation Unit; TF: trachomatous inflammation—follicular.

*Defined as present if the household respondent indicated that water used for washing faces during the dry season by household members was either (1) piped water into the dwelling, yard, or plot; or (2) any source located in the yard; or (3) any source where the time to go there, get water, and come back was <30 minutes.

^†^Defined as present if the household respondent indicated that adults in the household usually defecated in latrines (private, shared, or public) or other non-open sites (e.g. chamber pots, buckets, or in bodies of surface water).

^‡^Defined by the presence of ≥5 follicles, ≥0.5mm in diameter, in the central part of the upper tarsal conjunctiva in either eye.

^§^Reaching elimination target group includes EUs in which the most recent survey was an impact survey preceded by a previous impact or baseline survey that indicated that the EU had not met the elimination target of <5% prevalence of TF among children aged 1–9 years (TF_1-9_).

^‖^Maintaining elimination target group includes EUs in which the most recent survey was a surveillance survey preceded by a previous impact survey that indicated that the EU had met the elimination target of <5% prevalence of TF_1-9_.

Approximately half of the EUs in both groups had existing face-washing water coverages greater than 50% (S3 Fig). The latrine coverage distributions were skewed toward higher coverage, with the skew of the maintaining elimination target EUs distribution more pronounced (median 90%) than the reaching elimination target distribution (median 74%).

### Household exposures

Among children in both groups of EUs, household nearby face-washing water use and latrine use were associated with lower TF odds compared with households not meeting those criteria. In the reaching elimination target EUs, the odds ratio (OR) was 0.94 (95% CI: 0.90–0.99) for household use of nearby face-washing water and 0.85 (95% CI: 0.81–0.89) for household latrine use. In the maintaining elimination target EUs, the OR was 0.91 (95% CI: 0.84–0.98) for household nearby face-washing water use and 0.80 (95% CI: 0.74–0.87) for household latrine use. Parameter estimates from regression models can be found in S1 Table.

### EU-level hypothetical interventions: reaching elimination target EUs

The reaching elimination target EUs model included 423,000 examined children with complete covariate information. The model-derived TF_1-9_ in the observed data was 4.7% (the prevalence estimate from which absolute and relative changes below are calculated).

**Fig 2:**
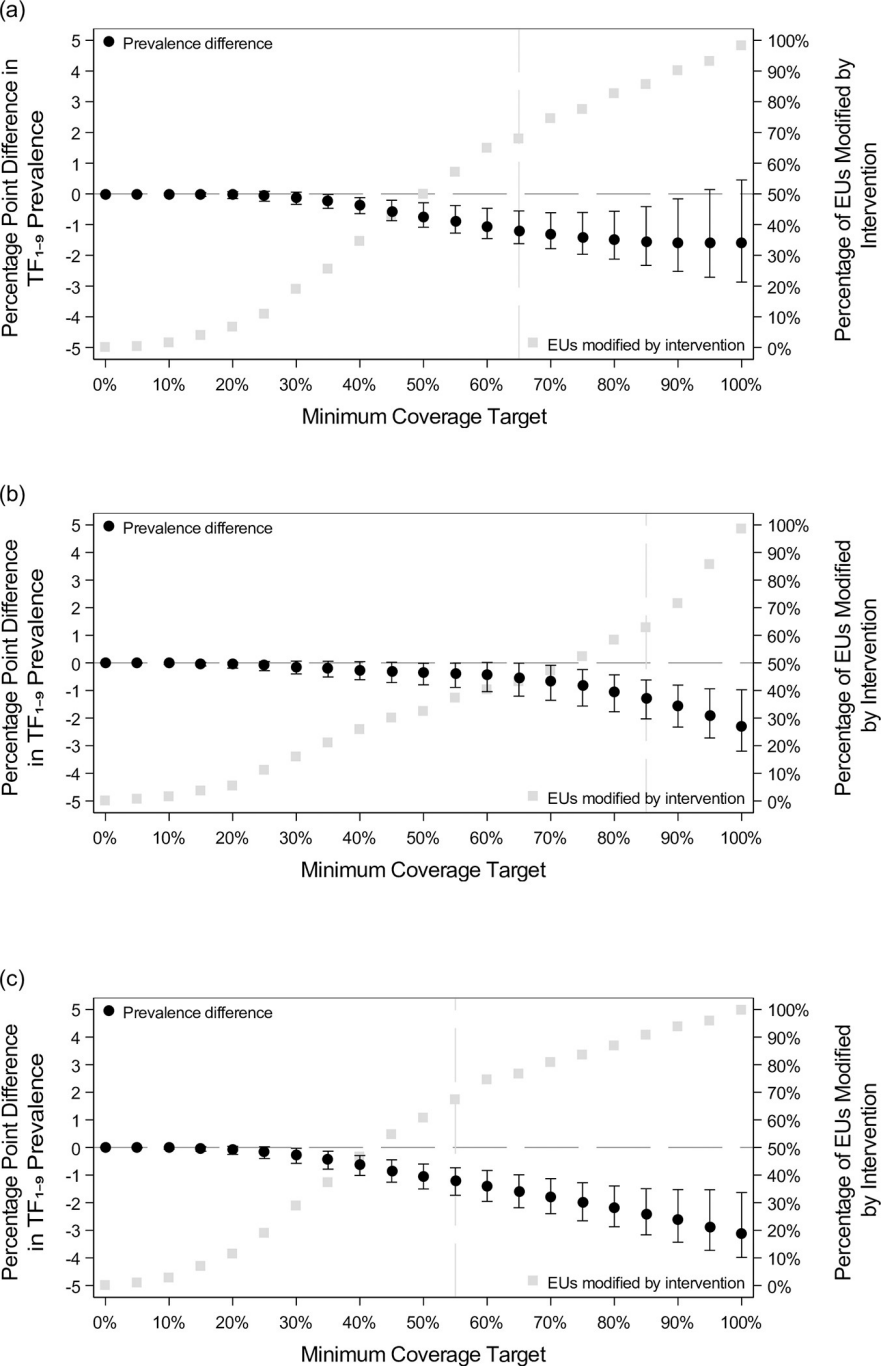
Prevalence difference and number and percentage of EUs modified after implementing hypothetical interventions on nearby face-washing water and latrine coverages among reaching elimination target EUs. These figures show the results presented in Table 2 graphically for (a) nearby face-washing water interventions, (b) latrine interventions, and (c) simultaneous interventions on both nearby face-washing water and latrines for the reaching elimination target EUs. When present, the vertical bar indicates the minimum coverage target at which the relative prevalence decrease was at least 25%.

For nearby face-washing water coverage interventions (Table 2 and Fig 2A), TF_1-9_ decreased negligibly until ~30% minimum coverage target was reached. TF_1-9_ then decreased in a linear-like manner until plateauing at a ~1.6 percentage points prevalence decrease at 85% minimum coverage target. A ≥25% reduction in TF_1-9_ was reached at 65% minimum coverage target (68% of EUs modified).

**Table 2:** TF_1-9_ prevalence difference and number and percentage of EUs modified after implementing hypothetical interventions on nearby face-washing water and latrine coverages.

Intervention implemented		Reaching elimination target EUs		Maintaining elimination target EUs	
Nearby face-washing water minimum coverage target	Latrine minimum coverage target	Prevalence difference*	EUs modified to reach minimum coverage target	Prevalence difference^†^	EUs modified to reach minimum coverage target
**Nearby face-washing water interventions**					
5%	None	0.0 (0.0, 0.0)	1 (0.3%)	0.0 (0.0, 0.0)	2 (0.8%)
10%	None	0.0 (0.0, 0.0)	5 (1.5%)	0.0 (0.0, 0.0)	5 (2.1%)
15%	None	0.0 (-0.1, 0.0)	13 (3.9%)	0.0 (0.0, 0.0)	10 (4.1%)
20%	None	0.0 (-0.2, 0.1)	22 (6.6%)	0.0 (-0.1, 0.0)	24 (10.0%)
25%	None	-0.1 (-0.2, 0.1)	36 (10.8%)	0.0 (-0.1, 0.0)	38 (15.8%)
30%	None	-0.1 (-0.3, 0.1)	63 (18.9%)	0.0 (-0.2, 0.0)	54 (22.4%)
35%	None	-0.2 (-0.5, 0.0)	85 (25.5%)	-0.1 (-0.2, 0.0)	71 (29.5%)
40%	None	-0.4 (-0.6, -0.1)	115 (34.5%)	-0.1 (-0.3, 0.0)	88 (36.5%)
45%	None	-0.6 (-0.9, -0.2)	143 (42.9%)	-0.2 (-0.4, 0.0)	106 (44.0%)
50%	None	-0.7 (-1.1, -0.3)	166 (49.8%)	-0.2 (-0.5, 0.0)	127 (52.7%)
55%	None	-0.9 (-1.3, -0.4)	190 (57.1%)	-0.3 (-0.5, 0.0)	144 (59.8%)
60%	None	-1.1 (-1.5, -0.5)	216 (64.9%)	-0.3 (-0.6, 0.0)	159 (66.0%)
65%	None	-1.2 (-1.6, -0.6)	226 (67.9%)	-0.3 (-0.6, 0.1)	173 (71.8%)
70%	None	-1.3 (-1.8, -0.6)	248 (74.5%)	-0.3 (-0.6, 0.2)	183 (75.9%)
75%	None	-1.4 (-2.0, -0.6)	258 (77.5%)	-0.2 (-0.6, 0.3)	188 (78.0%)
80%	None	-1.5 (-2.1, -0.6)	275 (82.6%)	-0.1 (-0.6, 0.6)	194 (80.5%)
85%	None	-1.6 (-2.3, -0.4)	285 (85.6%)	0.1 (-0.6, 0.9)	200 (83.0%)
90%	None	-1.6 (-2.5, -0.2)	300 (90.1%)	0.3 (-0.7, 1.4)	200 (83.0%)
95%	None	-1.6 (-2.7, 0.1)	310 (93.1%)	0.7 (-0.7, 2.3)	212 (88.0%)
100%	None	-1.6 (-2.9, 0.5)	327 (98.2%)	1.0 (-0.8, 3.2)	234 (97.1%)
**Latrine use interventions**					
None	5%	0.0 (0.0, 0.0)	2 (0.6%)	0.0 (0.0, 0.0)	0 (0.0%)
None	10%	0.0 (-0.1, 0.0)	5 (1.5%)	0.0 (0.0, 0.0)	0 (0.0%)
None	15%	0.0 (-0.1, 0.0)	12 (3.6%)	0.0 (0.0, 0.0)	3 (1.2%)
None	20%	0.0 (-0.2, 0.0)	18 (5.4%)	0.0 (0.0, 0.0)	5 (2.1%)
None	25%	-0.1 (-0.3, 0.0)	37 (11.1%)	0.0 (0.0, 0.1)	12 (5.0%)
None	30%	-0.1 (-0.4, 0.1)	53 (15.9%)	0.0 (0.0, 0.1)	18 (7.5%)
None	35%	-0.2 (-0.5, 0.1)	70 (21.0%)	0.0 (0.0, 0.2)	20 (8.3%)
None	40%	-0.3 (-0.6, 0.0)	86 (25.8%)	0.1 (0.0, 0.2)	25 (10.4%)
None	45%	-0.3 (-0.7, 0.0)	100 (30.0%)	0.1 (0.0, 0.3)	26 (10.8%)
None	50%	-0.4 (-0.8, 0.0)	108 (32.4%)	0.1 (0.0, 0.3)	30 (12.4%)
None	55%	-0.4 (-0.9, 0.0)	124 (37.2%)	0.1 (-0.1, 0.4)	32 (13.3%)
None	60%	-0.4 (-1.0, 0.0)	134 (40.2%)	0.1 (-0.1, 0.4)	41 (17.0%)
None	65%	-0.5 (-1.2, 0.0)	144 (43.2%)	0.1 (-0.1, 0.5)	45 (18.7%)
None	70%	-0.7 (-1.4, -0.1)	157 (47.1%)	0.1 (-0.1, 0.5)	49 (20.3%)
None	75%	-0.8 (-1.6, -0.2)	174 (52.3%)	0.1 (-0.1, 0.5)	57 (23.7%)
None	80%	-1.0 (-1.8, -0.4)	194 (58.3%)	0.1 (-0.2, 0.5)	69 (28.6%)
None	85%	-1.3 (-2.0, -0.6)	209 (62.8%)	0.1 (-0.3, 0.6)	85 (35.3%)
None	90%	-1.6 (-2.3, -0.8)	238 (71.5%)	0.0 (-0.4, 0.6)	122 (50.6%)
None	95%	-1.9 (-2.7, -0.9)	285 (85.6%)	-0.2 (-0.6, 0.4)	182 (75.5%)
None	100%	-2.3 (-3.2, -1.0)	328 (98.5%)	-0.7 (-1.1, 0.4)	240 (99.6%)
**Simultaneous interventions**					
5%	5%	0.0 (0.0, 0.0)	3 (0.9%)	0.0 (0.0, 0.0)	2 (0.8%)
10%	10%	0.0 (-0.1, 0.0)	9 (2.7%)	0.0 (0.0, 0.0)	5 (2.1%)
15%	15%	0.0 (-0.1, 0.0)	23 (6.9%)	0.0 (0.0, 0.0)	13 (5.4%)
20%	20%	-0.1 (-0.2, 0.0)	38 (11.4%)	0.0 (-0.1, 0.0)	28 (11.6%)
25%	25%	-0.1 (-0.4, 0.0)	63 (18.9%)	0.0 (-0.1, 0.1)	45 (18.7%)
30%	30%	-0.3 (-0.6, 0.0)	96 (28.8%)	0.0 (-0.2, 0.1)	64 (26.6%)
35%	35%	-0.4 (-0.8, -0.1)	124 (37.2%)	0.0 (-0.2, 0.1)	80 (33.2%)
40%	40%	-0.6 (-1.0, -0.3)	155 (46.5%)	-0.1 (-0.3, 0.1)	101 (41.9%)
45%	45%	-0.8 (-1.3, -0.4)	182 (54.7%)	-0.1 (-0.3, 0.1)	119 (49.4%)
50%	50%	-1.0 (-1.5, -0.6)	202 (60.7%)	-0.1 (-0.4, 0.1)	138 (57.3%)
55%	55%	-1.2 (-1.7, -0.7)	224 (67.3%)	-0.2 (-0.5, 0.2)	154 (63.9%)
60%	60%	-1.4 (-2.0, -0.8)	248 (74.5%)	-0.2 (-0.5, 0.2)	167 (69.3%)
65%	65%	-1.6 (-2.2, -1.0)	255 (76.6%)	-0.2 (-0.5, 0.3)	180 (74.7%)
70%	70%	-1.8 (-2.4, -1.1)	269 (80.8%)	-0.1 (-0.6, 0.4)	192 (79.7%)
75%	75%	-2.0 (-2.7, -1.3)	278 (83.5%)	-0.1 (-0.6, 0.5)	197 (81.7%)
80%	80%	-2.2 (-2.9, -1.4)	289 (86.8%)	0.1 (-0.6, 0.8)	204 (84.6%)
85%	85%	-2.4 (-3.2, -1.5)	302 (90.7%)	0.2 (-0.6, 1.1)	213 (88.4%)
90%	90%	-2.6 (-3.4, -1.5)	312 (93.7%)	0.4 (-0.7, 1.6)	217 (90.0%)
95%	95%	-2.9 (-3.7, -1.5)	319 (95.8%)	0.4 (-0.9, 1.9)	231 (95.9%)
100%	100%	-3.1 (-4.0, -1.6)	332 (99.7%)	0.0 (-1.2, 2.3)	241 (100.0%)

Figures shown in prevalence differences columns are the estimates and 95% confidence intervals. The prevalence difference is estimated as the mean predicted probability from the hypothetical intervention scenario minus the mean predicted probability from the observed data. Figures shown in the EUs modified columns are the count and percentage of EUs modified. EU: Evaluation Unit, TF_1-9_: trachomatous inflammation—follicular among children aged 1–9 years.

*Model-derived TF_1-9_ prevalence in the observed data was 4.7%.

^†^Model-derived TF_1-9_ prevalence in the observed data was 2.0%.

For latrine coverage interventions (Fig 2B), TF_1-9_ decreases were modest until reaching ~80% minimum coverage target, whereafter the estimated prevalence dropped sharply, with a 5-unit increase in coverage resulting in a ~0.3–0.4 percentage point TF_1-9_ decrease. At 85% minimum coverage target (63% of EUs modified) and above, we estimated a ≥25% decrease in prevalence.

When simultaneously applying interventions (Fig 2C), at 25% minimum coverage target, TF_1-9_ began to decrease in a nearly linear manner as coverage increased. A ≥25% decrease in prevalence was observed at 55% minimum coverage target (68% of EUs modified) and beyond.

### EU-level hypothetical interventions: maintaining elimination target EUs

**Fig 3:**
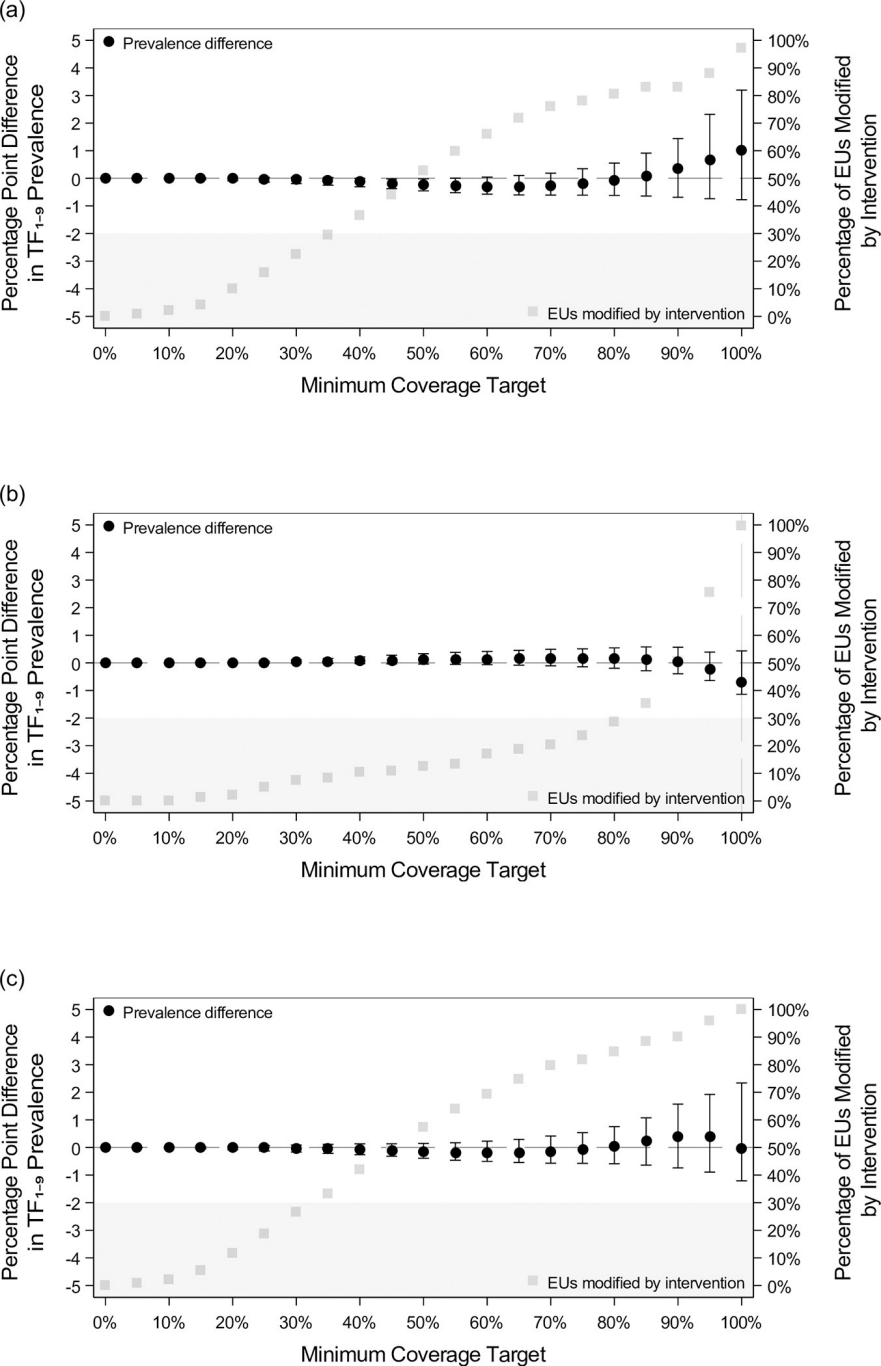
Prevalence difference and number and percentage of EUs modified after implementing hypothetical interventions on nearby face-washing water and latrine coverages among maintaining elimination target EUs. These figures show the results presented in Table 2 graphically for (a) nearby face-washing water interventions, (b) latrine interventions, and (c) simultaneous interventions on both nearby face-washing water and latrines for the maintaining elimination target EUs. When present, the vertical bar indicates the minimum coverage target at which the relative prevalence decrease was at least 25%. Shaded areas indicate values the prevalence difference cannot reach.

The maintaining elimination target EUs model included 330,971 examined children with complete covariate information. The model-derived TF_1-9_ in the observed data was 2.0% (the prevalence estimate from which absolute and relative changes below are calculated). Overall, results were less uniform in direction and less precise than those for the reaching elimination target EUs (Table 2 and Fig 3).

Increased nearby face-washing water coverages (Fig 3A) resulted in minimal changes in TF_1-9_ from 5% to ~85% minimum coverage target, at which point TF_1-9_ estimates began increasing.

For latrine coverage (Fig 3B), we estimated minimal change in prevalence through 90% minimum coverage target, corresponding to few EUs requiring interventions over the range. There was a modest decrease in TF_1-9_ only when the highest two minimum coverage target were reached.

Simultaneous interventions (Fig 3C) approximately delivered the sum of the prevalence changes in the non-simultaneous water and latrine interventions at each minimum coverage target.

## Discussion

We estimated expected changes in TF_1-9_ for a series of hypothetical WaSH-related interventions that might be expected when EUs with coverage below a specified target were brought up to the target over the observation period. Among EUs seeking to reach TF_1-9_ <5%, our model indicated that increasing EU-level minimum coverages to >30% of both household face-washing water with <30 minutes roundtrip collection time and household adult latrine use was associated with success. Among EUs that had previously met the TF_1-9_ elimination target, in which annual MDA distribution had been discontinued, results were mixed. Additional studies are needed to explore the WaSH-trachoma relationship in the latter context.

### Reaching elimination target EUs

In this group, the estimated decrease in TF_1-9_ when applying both water- and sanitation-related interventions is generally consistent with evidence generated from observational studies [20]. The pattern we observed as WaSH coverage increased differed by intervention. Changes to nearby face-washing water coverage resulted in larger prevalence decreases at lower minimum coverages than those from latrine coverage interventions. However, as upper minimum coverage levels were reached (≥85%), the prevalence decrease associated with face-washing water plateaued, while the latrine-related prevalence decrease accelerated. In previous work to identify thresholds (using a dataset that overlapped with ours), Garn *et al*. estimated a similar threshold for latrine use [21]. Our model extends Garn *et al*.’s [21] investigation by including data from serial surveys to improve confounding adjustment and model hypothetical changes in relevant exposures. Our finding is consistent with a belief that high latrine coverages may be needed to sufficiently reduce fly breeding sites to confer community-level protection, whereas household water use may more directly benefit individual households.

To illuminate potential resource needs, we estimated the percentage of EUs needing intervention to reach specified TF_1-9_ outcomes. Among reaching elimination target EUs, to achieve a ≥25% relative reduction in TF_1-9_ (~1 absolute percentage point), ~60–70% of EUs would need interventions in all scenarios, applied to varying numbers of households within each EU. While rudimentary, these estimates suggest that WaSH investments needed to appreciably modify TF_1-9_ may be substantial. More formal health economic studies are awaited.

### Maintaining elimination target EUs

Results were less uniform here. Of course, the magnitude of any TF_1-9_ decreases in this group could not exceed the observed prevalence of 2.0%, and, whereas the goal for the reaching elimination target EUs is to *decrease* TF_1-9_ to <5%, for the maintaining elimination EUs the goal is to *maintain* TF_1-9_ <5%. This could entail a decrease, no change, or even a small increase in TF_1-9_ over the surveillance period.

Unexpectedly, there was a suggestion of a change in trend at high coverage of nearby face-washing water following hypothetical intervention. Statistical uncertainty (as evidenced by wide confidence intervals) precludes us from having assurance in this result, for which contributory data were relatively sparse. If the association is real, our (speculative) explanations would be as follows. First, we used a face-washing water exposure definition of <30-minute roundtrip collection time. Both behavioral plausibility and previous research [22,23] suggest that on-plot water use may be more strongly connected to disease outcomes because it confers considerably greater access to water for personal hygiene. Nearby face-washing water exposure may have sufficiently captured effects in the reaching elimination target EUs; however, in a group in which gains are expected to be small, the nearby exposure definition may be inadequate. Second, unmeasured confounding may be present. In particular, densely-populated areas with more economic activity might have high water coverage. Without MDA, these areas may be at increased risk of *C*. *trachomatis* reintroduction resulting from population movement. While we adjusted for population density, we did not directly capture migration.

For latrine coverage, there was minimal change in TF_1-9_ until the uppermost coverage levels were reached. Existing coverage was generally high, so it was unsurprising that little change in TF_1-9_ was seen across much of the coverage range. Unlike face-washing water, our findings provide some evidence that achieving very high latrine coverage (≥95%) could provide additional benefits, although results are imprecise. This finding supports the Sustainable Development Goal target of universal elimination of open defecation, rather than incremental improvements in sanitation coverage often found with sanitation interventions [24].

#### Household and community protection

Though ORs contrasting presence/absence of nearby face-washing water and latrine use at household level were similar for the two groups, we estimated that interventions would only be useful to reduce TF_1-9_ in EUs that had not yet met elimination targets. The overall intervention impacts depended on both individual-level effects, and the "contextual" effects of overall coverage in each EU [25]. Individual-level effects of interventions on infectious diseases may not translate to population-level effects, though more work is needed to understand why contextual effects might differ across these two groups, and whether contextual effects may be better captured at scales other than the EU level.

### Limitations

First, the relationship between face-washing water use and active trachoma may be best evaluated using on-plot water. This was our preferred definition; however, the median on-plot face-washing water use coverage in both groups was <5%; thus, that analysis was precluded by sparse data. Our use of nearby face-washing water likely dampened associations in this analysis. Future studies may be informative as on-plot water availability increases in these communities [26,27]. Second, our hypothetical intervention randomly allocated exposures at cluster and household levels. While we designed interventions that could be both testable in a trial and scalable in the real world, we acknowledge that random allocation may be both difficult to implement and sub-optimal in effect. For example, while providing WaSH interventions only to households with children could accelerate TF_1-9_ reductions, it may raise ethical challenges. Third, we use the term "intervention" to describe any hypothetical approach or combination of approaches that would achieve the desired minimum coverage levels. We assume that the delivery mechanism (e.g., engineering, climate, public health program) would not directly influence the impact of increased WaSH access. Fourth, the effectiveness of washing water for reducing facial *C*. *trachomatis* is likely to depend on factors beyond the distance to a water source, including the efficiency of washing practices, the time of year (e.g., dry vs. wet season) [26], competing priorities (e.g., household or agricultural duties), and cultural practices. Finally, study results may not be generalizable to countries outside those that contributed data, as trachoma epidemiology and WaSH availability and use may substantially differ.

Here, we provide an approach that frames flexible multilevel models in terms of interpretable, population-level associations. As with any data analysis, to infer causality, we must assume (among other things) that the identifiability conditions of *consistency* and *conditional exchangeability with positivity* are met [27]. Above, using language geared toward readers less familiar with causal inference terminology, we allude to a potential violation of consistency. We felt it reasonable to assume, however, that how a person came to use a latrine for defecation is unlikely have an independent impact on the outcome. In the case of face washing water use, where rainfall alone may contribute to water use or water use may increase due to active involvement of health care and governmental organizations, this assumption may be more suspect due to differing anticipated side-effects. As with all observational studies, violations of exchangeability (i.e., unmeasured confounding) are possible and cannot be verified with our data. We believe this study, however, provides improved adjustment as we incorporated historical WaSH coverages and population density into our models. While we made best efforts to achieve the identifiability conditions, whether they have been achieved can (and should) be debated to improve causal inference in WaSH research by means of more expansive data collection.

### Conclusions

We used existing data to estimate population-level impacts of increasing WaSH coverage on reducing TF_1-9_. Our approach is closely tied to policy-setting goals and allowed us to approximate the expected direction and magnitude of association. The Neglected Tropical Disease Road Map 2021–2030 may energize both the WaSH and trachoma communities with its call for increased collaboration [28,29]. Our results provide researchers with data to inform future studies that could ultimately enhance the effectiveness of SAFE’s "E" component.

## Supporting information

S1 STROBE Checklist STROBE checklist. (DOCX)

S1 Fig Directed acyclic graph showing the assumed relationships between water, sanitation, and hygiene factors and the presence of trachomatous inflammation—follicular among children aged 1–9 years. EU: Evaluation Unit; HH: Household; The time of the most recent survey is denoted with t = 2 and the time of the prior survey is denoted with a t = 1; t = 2–1 indicates the interval between the survey periods; created at http://dagitty.net/.
(TIF)

S2 Fig Abbreviated description of G-computation steps used in this analysis. (TIF)

S3 Fig EU-level distributions of nearby face-washing water and latrine coverages by elimination group. Among the reaching elimination target EUs, the coverage distributions of (a) nearby face-washing water and (b) latrine use are shown. Among the maintaining elimination target EUs, the coverage distributions of (c) nearby face-washing water and (d) latrine use are shown. The lower bound of each bin corresponds to the hypothetical intervention minimum coverage targets evaluated in the analysis.
(TIF)

S1 Table Results from the logistic hierarchical models by elimination group. (DOCX)
