# Supplementary figures and images for: Exploring water, sanitation, and hygiene coverage targets for reaching and sustaining trachoma elimination: G-computation analysis
Source: PLoS Negl Trop Dis. 2023 Feb 13;17(2):e0011103. doi: 10.1371/journal.pntd.0011103 (PMC9925017; doi:10.1371/journal.pntd.0011103)

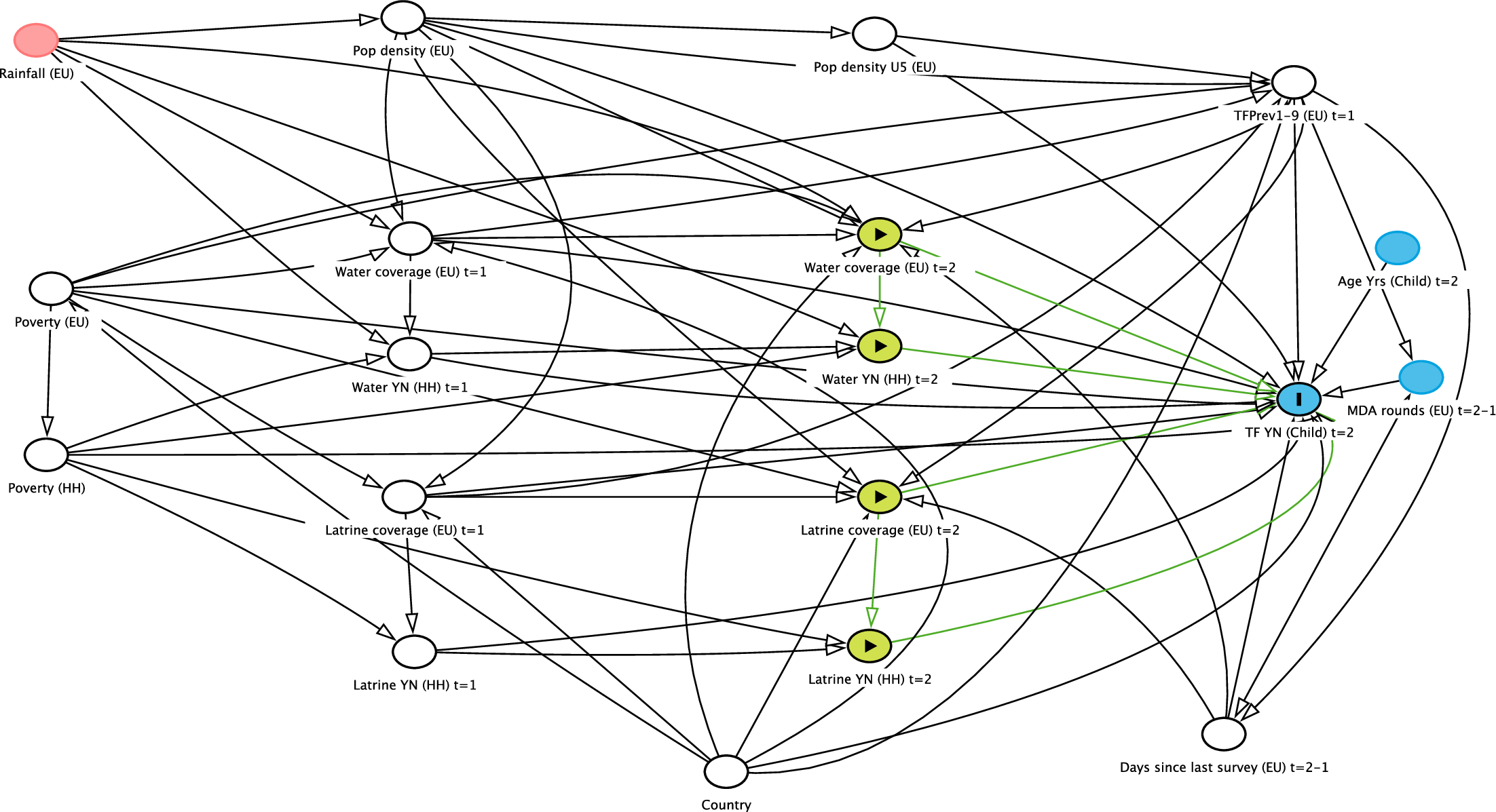

Supplement: S1 Fig — EU: Evaluation Unit; HH: Household; The time of the most recent survey is denoted with t = 2 and the time of the prior survey is denoted with a t = 1; t = 2–1 indicates the interval between the survey periods; created at http://dagitty.net/. (TIF) [file pntd.0011103.s002.tif]

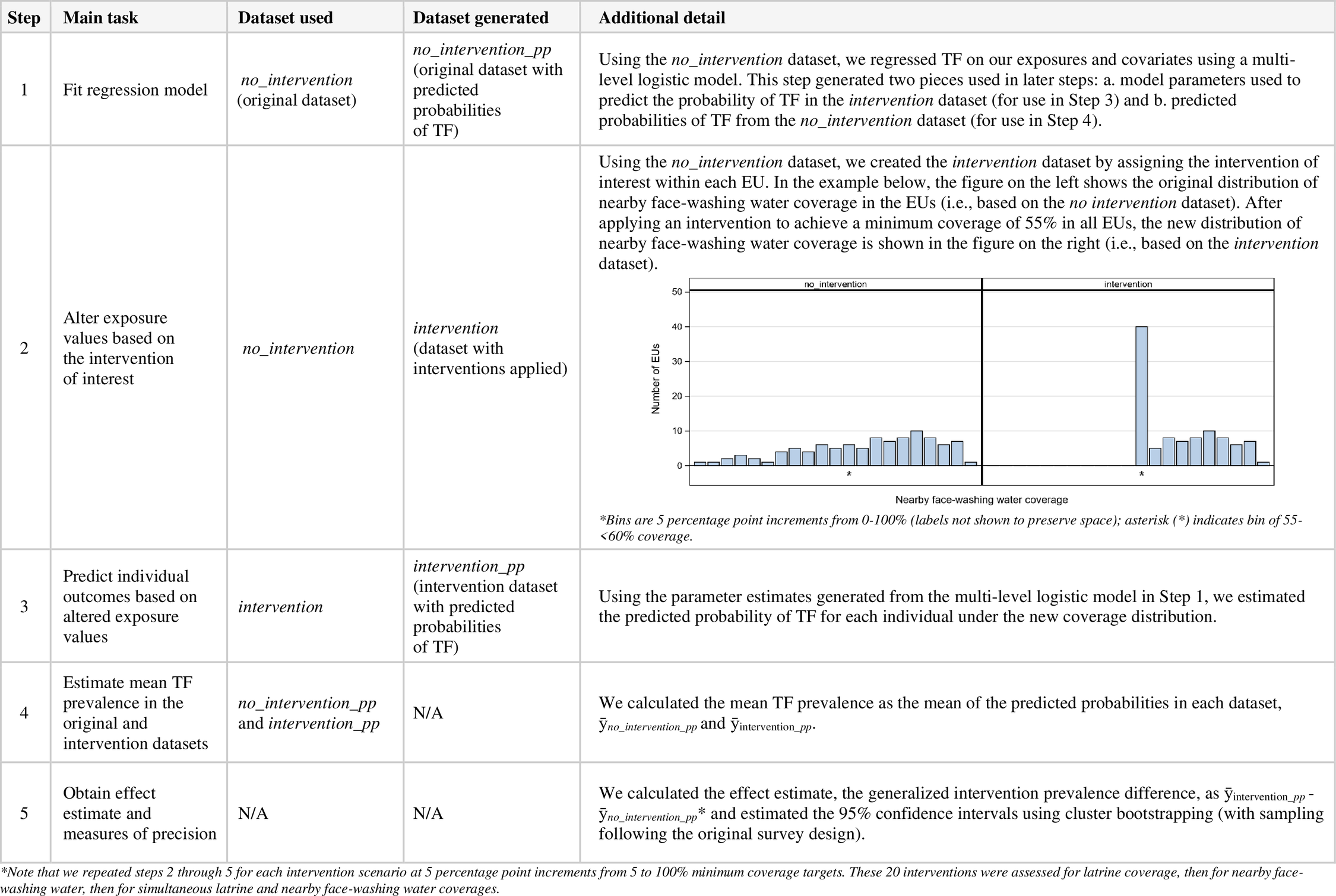

Supplement: S2 Fig — (TIF) [file pntd.0011103.s003.tif]

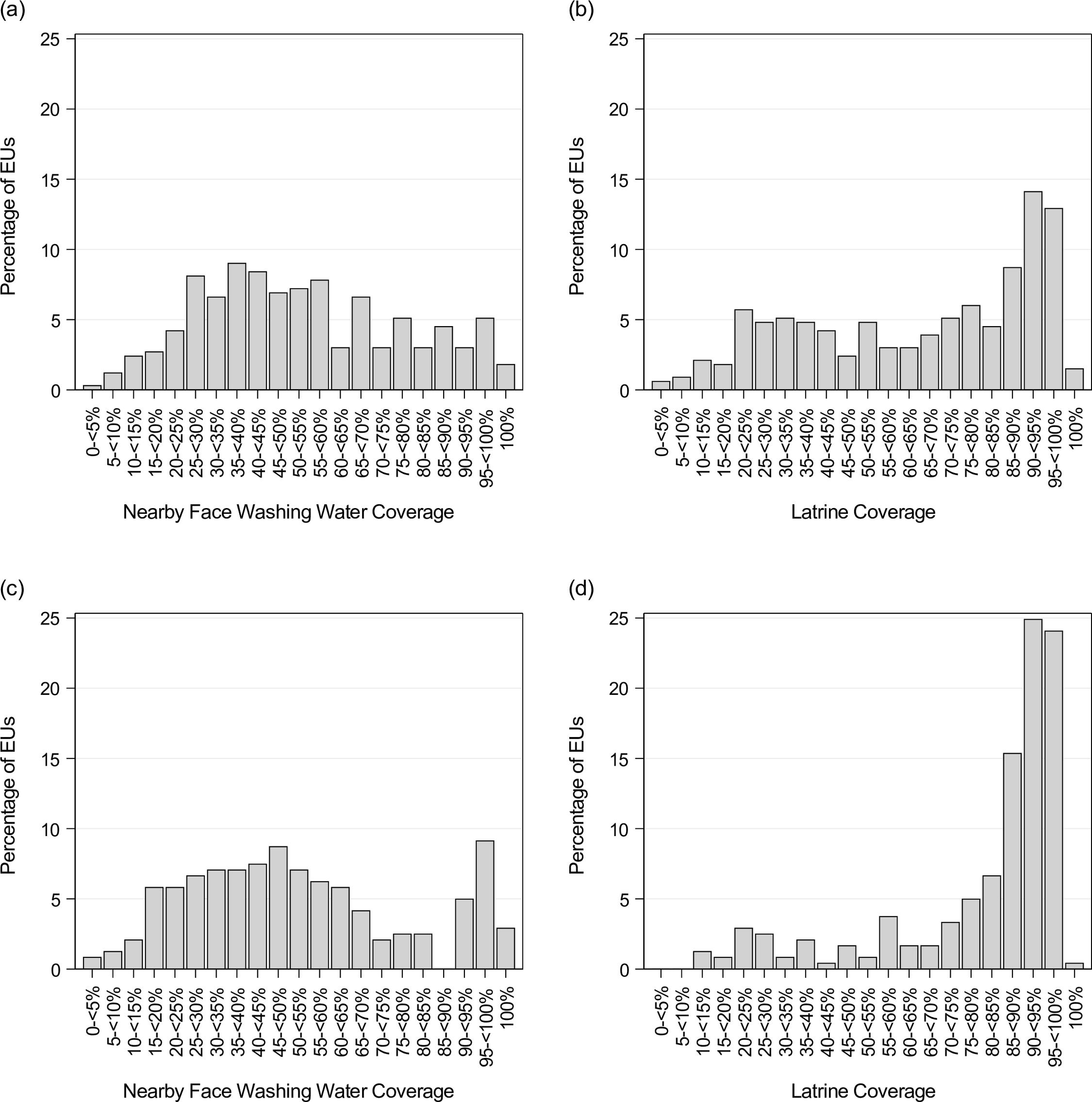

Supplement: S3 Fig — Among the reaching elimination target EUs, the coverage distributions of (a) nearby face-washing water and (b) latrine use are shown. Among the maintaining elimination target EUs, the coverage distributions of (c) nearby face-washing water and (d) latrine use are shown. The lower bound of each bin corresponds to the hypothetical intervention minimum coverage targets evaluated in the analysis. (TIF) [file pntd.0011103.s004.tif]
