# Supplementary material for: Exploring water, sanitation, and hygiene coverage targets for reaching and sustaining trachoma elimination: G-computation analysis
Source: PLoS Negl Trop Dis. 2023 Feb 13;17(2):e0011103. doi: 10.1371/journal.pntd.0011103 (PMC9925017; doi:10.1371/journal.pntd.0011103)
Supplement: S1 Table — (DOCX) [file pntd.0011103.s005.docx]

|  | **Reaching elimination target EUs model:^*^**  **estimate (standard error)** | **Maintaining elimination target EUs model:^†^**  **estimate (standard error)** |
| --- | --- | --- |
| **Random error variance** |  |  |
| EU (level-3 intercept) | 0.49 (0.05) | 0.26 (0.04) |
| Cluster (level-2 intercept) | 1.11 (0.03) | 1.06 (0.05) |
| **Fixed effects**^‡^ |  |  |
| Intercept | -0.84 (0.67) | 2.83 (2.60) |
| Presence of household nearby  face-washing water | -0.06 (0.02) | -0.10 (0.04) |
| EU nearby face-washing coverage | 0.00 (0.02) | 0.00 (0.01) |
| EU nearby face-washing coverage (basis function 1) | -0.03 (0.03) | -0.02 (0.02) |
| EU nearby face-washing coverage (basis function 2) | 0.04 (0.04) | 0.06 (0.03) |
| Presence of household latrine use | -0.16 (0.02) | -0.22 (0.04) |
| EU latrine coverage | -0.02 (0.01) | 0.02 (0.01) |
| EU latrine coverage (basis function 1) | 0.02 (0.02) | -0.01 (0.01) |
| EU latrine coverage (basis function 2) | -0.05 (0.04) | -0.22 (0.12) |
| EU nearby face-washing coverage -  prior survey | -0.03 (0.02) | 0.02 (0.01) |
| EU nearby face-washing coverage -  prior survey (basis function 1) | 0.05 (0.03) | -0.02 (0.02) |
| EU nearby face-washing coverage -  prior survey (basis function 2) | -0.07 (0.04) | 0.00 (0.03) |
| EU latrine coverage - prior survey | 0.00 (0.01) | -0.02 (0.01) |
| EU latrine coverage - prior survey (basis function 1) | 0.02 (0.02) | 0.03 (0.01) |
| EU latrine coverage - prior survey (basis function 2) | -0.06 (0.04) | 0.05 (0.06) |
| EU TF_1-9_ prevalence - prior survey | 0.10 (0.04) | 0.12 (0.18) |
| EU TF_1-9_ prevalence - prior survey (basis function 1) | -0.24 (0.10) | 0.20 (0.24) |
| EU TF_1-9_ prevalence - prior survey (basis function 2) | 0.30 (0.12) | -0.43 (0.45) |
| Age | 0.40 (0.03) | 0.59 (0.04) |
| Age (basis function 1) | -0.86 (0.04) | -1.02 (0.07) |
| Age (basis function 2) | 0.84 (0.06) | 0.99 (0.10) |
| Household population density (log) | -0.61 (0.12) | 0.00 (0.14) |
| Household population density (log) (basis function 1) | 0.37 (0.16) | -0.50 (0.23) |
| Household population density (log) (basis function 2) | -0.83 (0.33) | 0.43 (0.41) |
| Years between surveys | -0.33 (0.15) | -4.23 (1.27) |
| Years between surveys (basis function 1) | 0.17 (0.17) | 5.03 (2.17) |
| Years between surveys (basis function 2) | 1.42 (0.95) | -5.18 (2.57) |
| Benin | -2.27 (0.40) | -- |
| Burundi | -0.55 (0.78) | -- |
| Cameroon | --^§^ | 1.18 (0.27) |
| Ethiopia | reference | 0.56 (0.24) |
| Guinea-Bissau | -- | 0.36 (0.38) |
| Malawi | -1.31 (0.24) | -0.18 (0.18) |
| Mauritania | -- | -1.45 (0.41) |
| Mozambique | -0.74 (0.22) | 0.24 (0.19) |
| Niger | -- | 1.06 (0.46) |
| Nigeria | -0.70 (0.21) | 0.36 (0.30) |
| Senegal | -0.66 (0.38) | -0.52 (0.34) |
| Sudan | -0.33 (0.43) | -- |
| Tanzania | -0.61 (0.22) | reference |
| Uganda | -0.91 (0.31) | -0.66 (0.16) |
| Yemen | 0.27 (0.45) | -- |
| Zambia | -0.34 (0.31) | -0.10 (0.34) |

^*^423,000 examined children ages 1-9 years old with complete covariate information were included in model.

^†^330,971 examined children ages 1-9 years old with complete covariate information were included in model.

^‡^All continuous variables were modeled as restricted quadratic splines with knots placed at the 5^th^, 50^th^, and 95^th^ percentiles of the case distributions.

^§^Symbol ‘--’ indicates that the country was not included in the model because there were no eligible EUs.
